# Suicide attempt using sodium nitrite ordered on the internet: Two case reports

**DOI:** 10.1097/MD.0000000000029355

**Published:** 2022-07-15

**Authors:** Jae Chol Yoon, So Eun Kim

**Affiliations:** a Research Institute of Clinical Medicine of Jeonbuk National University and Biomedical Research Institute of Jeonbuk National University Hospital, Jeonju-si, Republic of Korea; b Department of Emergency Medicine, Jeonbuk National University Medical School, Jeonju, Republic of Korea.

**Keywords:** intentional, methemoglobinemia, sodium nitrite, suicide, toxicity

## Abstract

**Rationale:**

Sodium nitrite is a potent oxidizing agent that impairs oxygen transport and delivery through methemoglobin formation. Clinical manifestations are known to induce methemoglobinemia, dysrhythmia, hypotension, and even death. While accidental intoxication of sodium nitrite by contaminated water and food has previously occurred, there has been a substantial upsurge in suicide intoxication in recent years.

**Patient concerns:**

We present case reports of 2 patients who attempted suicide by sodium nitrite after ordering a “suicide powder” on the internet market. They were brought to the emergency department after attempting suicide by ingesting sodium nitrite. They experienced dyspnea, cyanosis, and mild nausea.

**Diagnosis:**

Based on their history and blood tests, methemoglobinemia was initially diagnosed.

**Interventions and outcomes:**

The patients received methylene blue antidotal therapy in the emergency department. The patients were discharged after neuropsychiatric evaluation and treatment for mental illness, suicidal ideation, and suicide attempts. They informed us of how simple and easy it was for them to buy sodium nitrite for suicidal purposes.

**Lessons:**

With widely shared information on the usage of sodium nitrite for suicide and the absence of proper regulation, the incidence of acute poisoning will increase. This increases physicians’ chances of encountering unexplained cyanosis and methemoglobinemia. Clinical suspicion of sodium nitrite intoxication is warranted in cases of unexplained cyanosis or methemoglobinemia. We want to highlight how simple and easy it is to buy sodium nitrite for suicidal purposes.

## 1. Introduction

Sodium nitrite (NaNO_2_) is an easily accessible colorless inorganic compound. It is used as a preservative, color fixative, and antimicrobial agent, mainly in cheese, meat (such as ham, bacon, and sausages), and fish.^[1,2]^ Another use is as an industrial chemical and therapeutic antidote agent for cyanide intoxication.^[3]^ The primary toxicity mechanism of sodium nitrite is the oxidation of ferrous iron (Fe2+) of hemoglobin to methemoglobin (MetHb) containing ferric iron (Fe3+) with consequent methemoglobinemia, in which it loses its ability to bind and transport oxygen.^[4]^

While accidental intoxication by contaminated water and food has been widely reported in the past, intentional intoxication has rarely been described in the scientific literature.^[5]^ However, surprisingly, the number of suicides using sodium nitrite is increasing, mainly in 2021.^[6–8]^ Currently, in many countries, lethal amounts of sodium nitrite can be purchased for only a few dollars through online sales without proper regulation.^[8]^ We present case reports of 2 patients who attempted suicide using sodium nitrite ordered on the internet. The patients provided written informed consent for publication of this case report.

## 2. Case description

### 2.1. Case 1

**Figure 1.**
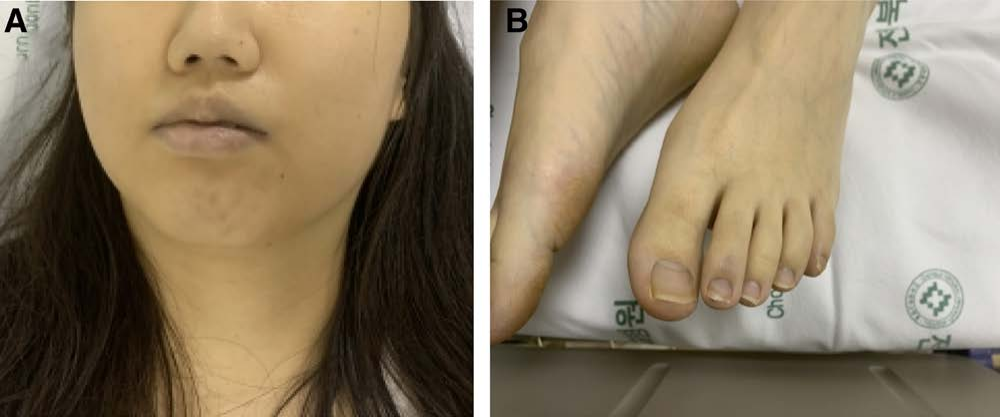
Cyanosis observed on the patient’s face and lip (A) and extremities and nail beds (B).

A 24-year-old previously healthy female intentionally ingested approximately 10 g of powdered sodium nitrite in a suicide attempt, following the instructions she obtained from a suicide blog. She recently felt depressed, was unable to work, and had trouble sleeping. She described her distress as increasing over the course of a few days, and she finally made plans to commit suicide. She purchased sodium nitrite online without difficulty or the need for any permissions. However, she stated that she regretted her decision immediately after ingesting sodium nitrite. She confessed to her family member that she was attempting suicide. She was admitted to the emergency department (ED) by her mother, 30 minutes after ingestion with acute dyspnea and cyanosis (Fig. 1).

**Figure 2.**
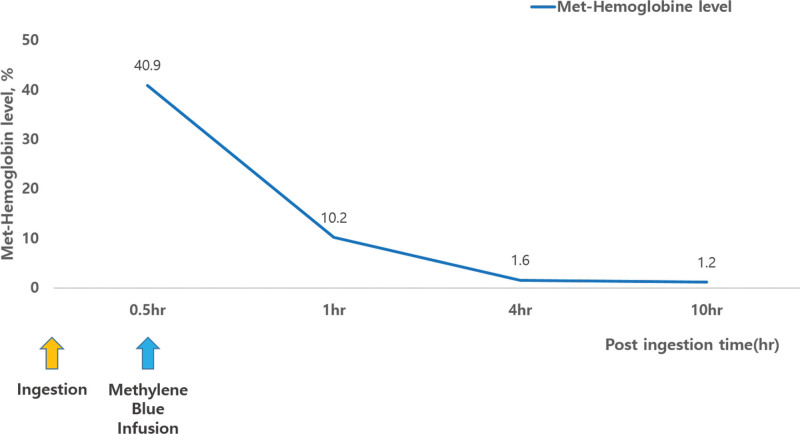
Methemoglobin level. Within 1 hour of methylene blue administration, methemoglobin levels decreased to 10.2% and then to 1.6% four hours later.

Upon admission, her vital signs were as follows: blood pressure, 117/54 mm Hg; pulse, 104 beats/min; respiratory rate, 24 breaths/min; and oxygen saturation, 93% on room air. Her initial methemoglobin level was 40.9%, and she was treated with a total dose of 1.0 mg/kg intravenous methylene blue. Within 1 hour of methylene blue administration, her symptoms improved significantly, and methemoglobin levels decreased to 10.2% and then to 1.6% four hours later (Fig. 2). The patient experienced no complications throughout her hospital stay. She was discharged after neuropsychiatric evaluation and treatment for mental illness, suicidal ideation, and suicide attempts.

### 2.2. Case 2

**Figure 3.**
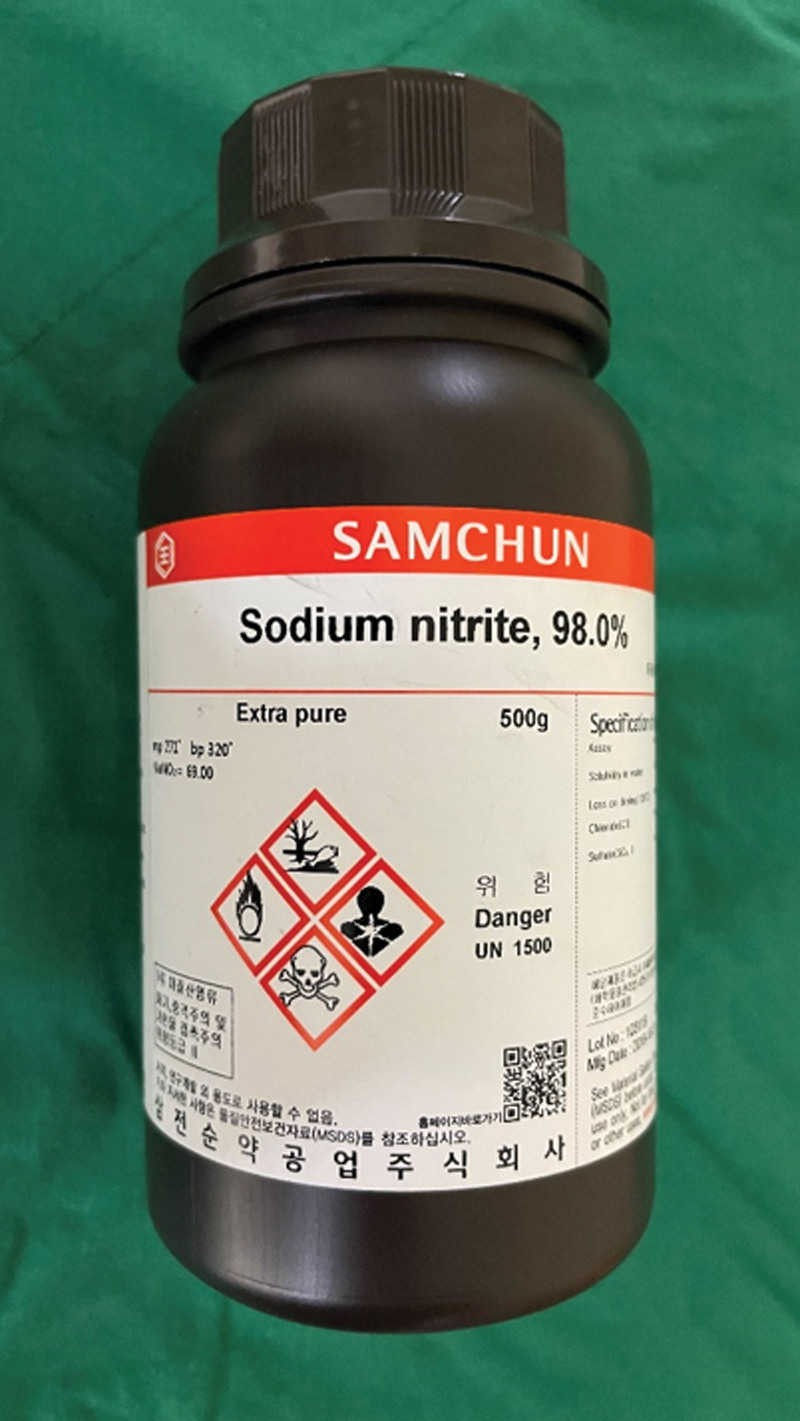
Sodium nitrite bottle, containing 500 mg of 98.0% sodium nitrite by weight. A teaspoonful of sodium nitrite has the potential to be lethal.

A 20-year-old man presented to the ED after intentional ingestion of 750 mg of powdered sodium nitrite (98% by weight). He had been experiencing major depressive disorder for the last 6 months but had not taken medicine recently. He was under stress from a college issue and felt pressured to succeed in the future. He remained depressed for most of the day and repeatedly thought of death. He prepared a pill by filling an empty pill capsule with sodium nitrite powder and taking it with water at home. One hour later, the patient visited the ED. He stated that he obtained information on how to make the suicide pill from an online suicide forum and purchased sodium nitrite online. He brought a bottle containing 500 grams of 98% sodium nitrite by weight that he purchased online (Fig. 3). He experienced only mild nausea a few minutes after ingesting a self-produced suicide pill. A physical examination revealed no signs of central or peripheral cyanosis, and the patient was hemodynamically stable. The initial methemoglobin level was 4.8. His methemoglobin level returned to normal 2 hours after ingestion. Methemoglobin levels did not change following repeated measurements. The patient recovered without any problems with conservative supportive treatment.

## 3. Discussion

Here, we report 2 unusual cases of intoxication caused by deliberate ingestion of sodium nitrite purchased online in the last 6-month period. The individuals confessed that they had learned about this suicide method in online forums and blogs. We searched the internet for “sodium nitrite” and “suicide” and found that several websites provide step-by-step instructions for suicide with sodium nitrite at home. They advertised sodium nitrite as an ideal suicide method, describing it as an “easy, quick, and painless recipe.”

While nonintentional intoxications with sodium nitrite have been frequently reported, sodium nitrite was rarely used for suicide prior to 2010. Worrisomely suicide cases using sodium nitrite have increased after detailed explanations were introduced in The Peaceful Pill Handbook, a suicide manual written by Australian doctors in 2017.^[9]^ Although printed books have been banned, they remain widely disseminated worldwide. In the book, sodium nitrite is outlined and advocated as a nonviolent and peaceful suicide method owing to its availability, preparation, undetectability, speed, safety, low price, and storage.^[9]^ Although in a few countries the sale of pure sodium nitrite is strictly controlled by state authorities, its free sale is prohibited, and it cannot be legally purchased for domestic use.^[4]^ However, in many countries, lethal amounts of sodium nitrite can be purchased for only a few dollars through the online open market without violating any regulations.^[8]^ Some websites advertise sodium nitrite as an ideal suicide agent and explain how to use it in detail. The rapid spread of information through social networking services (SNSs) and the internet has led to a sharp increase in the number of suicide attempts using sodium nitrite.^[10–12]^ Additionally, the number of web forums and shadow markets devoted to explaining how to use sodium nitrite for euthanasia is increasing. Discussion of the use of sodium nitrite for suicide is readily accessible anywhere in the world.^[13]^ There has been an increase in awareness in the scientific literature on the suicidal usage of sodium nitrite in many countries.^[6–8,10–12]^ These cases warn of the grim reality that there may be numerous additional suicide cases not reported in the scientific literature.

The lethal dose of sodium nitrite in adults is not precise, with a broad range reported between 0.7 and 6 g of nitrite component.^[6]^ However, based on the therapeutic dose of sodium nitrite used in cyanide poisoning in a typical adult, the lethal dose is approximately 2.6 g.^[3]^ This means that even a teaspoonful of sodium nitrite has the potential to be lethal. The latest edition of The Peaceful Pill Handbook, recommends that 20 g of sodium nitrite (approximately 1 spoon) should be used for suicide.^[9]^

Nitrites induce toxicity through the oxidation of ferrous iron (Fe2+) to ferric iron (Fe3+) in hemoglobin, producing methemoglobin. Blood containing methemoglobin is classically expressed as “chocolate brown” in appearance and is often recognized as an abnormality during blood sampling. Unlike normal hemoglobin, methemoglobin does not bind oxygen, resulting in functional anemia, diminished oxygen delivery to tissues, and the development of lactic acidosis. The oxidizing properties of sodium nitrite can also cause hemolysis, further impairing oxygen delivery. Finally, nitrites also act as potent vasodilators in the peripheral vasculature, resulting in vasodilatory shock.^[14]^

Methylene blue is the first-line antidote therapy for acute toxic methemoglobinemia with methemoglobin levels of >30%. Methylene blue is also appropriate for those who are symptomatic with methemoglobin levels between 20% and 30%, especially those with high-risk factors such as anemia, or pulmonary or cardiac comorbidities. It is administered at a dose of 1 to 2 mg/kg body weight intravenously over 5 minutes.^[15]^ Most cases show rapid clinical improvement with methylene blue and reduction of methemoglobin levels to <10% within 10 to 60 minutes. It may be repeated within 1 hour if the methemoglobin level remains high (e.g., >20%) and/or is increasing. Rapid diagnosis and early intervention with methylene blue infusion can prevent a fatal outcome as in the present case with an initial methemoglobin level of 92.5%.^[16]^ As shown in our first case, even if a lethal dose of sodium nitrite was taken, fatal outcomes can be prevented if early diagnosis and methylene blue treatment are started immediately.

However, administration of >2 to 3 doses (>7 mg/kg) is generally avoided because of the possibility of hemolysis.^[17]^ Alternative treatments include RBC or exchange transfusions to replace dysfunctional hemoglobin and hyperbaric oxygen therapy.^[18]^

Due to its wide application, controlling or monitoring the sale of sodium nitrite can be highly difficult. There is no proper regulation for sodium nitrite, and it is easy to find sodium nitrite for sale by internet vendors or any local laboratory supplier in relatively high quantities.

With widely shared information on the suicide usage of sodium nitrite and the absence of proper regulation, the incidence of acute poisoning will also increase. This increases physicians’ chances of encountering unexplained cyanosis and methemoglobinemia. A clinical suspicion of sodium nitrite intoxication would be warranted in cases of unexplained cyanosis or methemoglobinemia.

This is the first scientific report of suicide survivors using sodium nitrite in Korea. Survivors informed us of how simple and easy it was for them to buy sodium nitrite for suicide. We want to raise clinical awareness of sodium nitrite as a potential suicide method that induces methemoglobinemia and cyanosis to urge the government to intervene and monitor the sodium nitrite market.

## Author contributions

Conseptualization: Y.J.C.

Supervision: K.S.E.

Writing original draft: K.S.E.

Writing review & editing: Y.J.C., K.S.E.
